# A rare cause of partial small bowel obstruction

**DOI:** 10.1055/a-2719-3226

**Published:** 2025-11-05

**Authors:** Ben-Hua Wu, Jia-Lin Yuan, Li-Sheng Wang, Wen-Biao Chen

**Affiliations:** 1 12387 Department of Gastroenterology, Shenzhen Peopleʼs Hospital, Shenzhen, China; 2 12387 Department of Radiology, Shenzhen Peopleʼs Hospital, Shenzhen, China

A 59-year-old man presented with a five-month history of recurrent abdominal pain and vomiting. Five months prior to admission, during an episode of abdominal pain, an abdominal computed tomography (CT) revealed segmental wall thickening and blurring of the small bowel wall in the right mid-abdomen, with obscuration of the adjacent mesenteric fat planes. Mild upstream small bowel dilatation with fecalized content and mural blurring suggested partial small bowel obstruction ( Fig. 1 ). He was managed conservatively at a local hospital with fasting, intravenous fluids, and antibiotic therapy, which resulted in symptomatic improvement. However, he subsequently experienced two milder episodes of abdominal pain, neither of which received a medical evaluation.

**Fig. 1:**
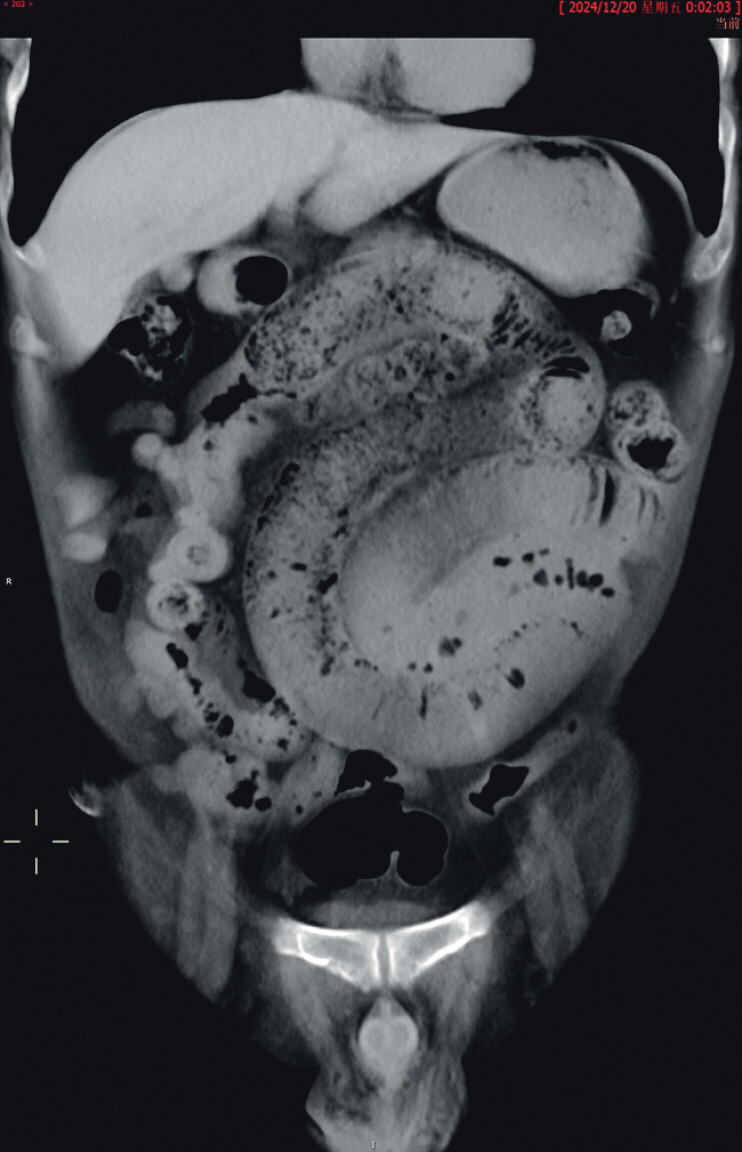
The computed tomography imaging manifestations suggest incomplete intestinal obstruction.

On admission, a physical examination revealed a soft, non-distended, and non-tender abdomen. Laboratory investigations revealed a normal white blood cell count (6.87 × 10^9/L) with mild eosinophilia (14.3%). No abnormalities were found in other blood biochemical tests. Upper gastrointestinal endoscopy revealed a nonspecific gastroduodenal inflammation. Random colonoscopic biopsy results were unremarkable. During oral double-balloon enteroscopy, advancement to approximately 300 cm into the ileum revealed a segment of markedly edematous and hyperemic small-bowel mucosa ( Fig. 2 **a** ). An actively motile live worm surrounded by congested and edematous mucosa with mild hemorrhage was observed penetrating the intestinal mucosa ( Fig. 2 **b** ). The helminth was successfully extracted using the biopsy forceps ( Fig. 2 **c** , Video 1 ). Histopathological analysis confirmed that the specimen was *Enterobius vermicularis* ( Fig. 3 **a–c** ). Based on this, the patient was clearly diagnosed with partial small bowel obstruction caused by enterobiasis of the small intestine. The patient underwent a course of albendazole, an anthelmintic agent effective against *Enterobius vermicularis* . Following antiparasitic therapy, the patient achieved complete resolution of symptoms, with no further episodes of abdominal pain or vomiting.

**Fig. 2:**
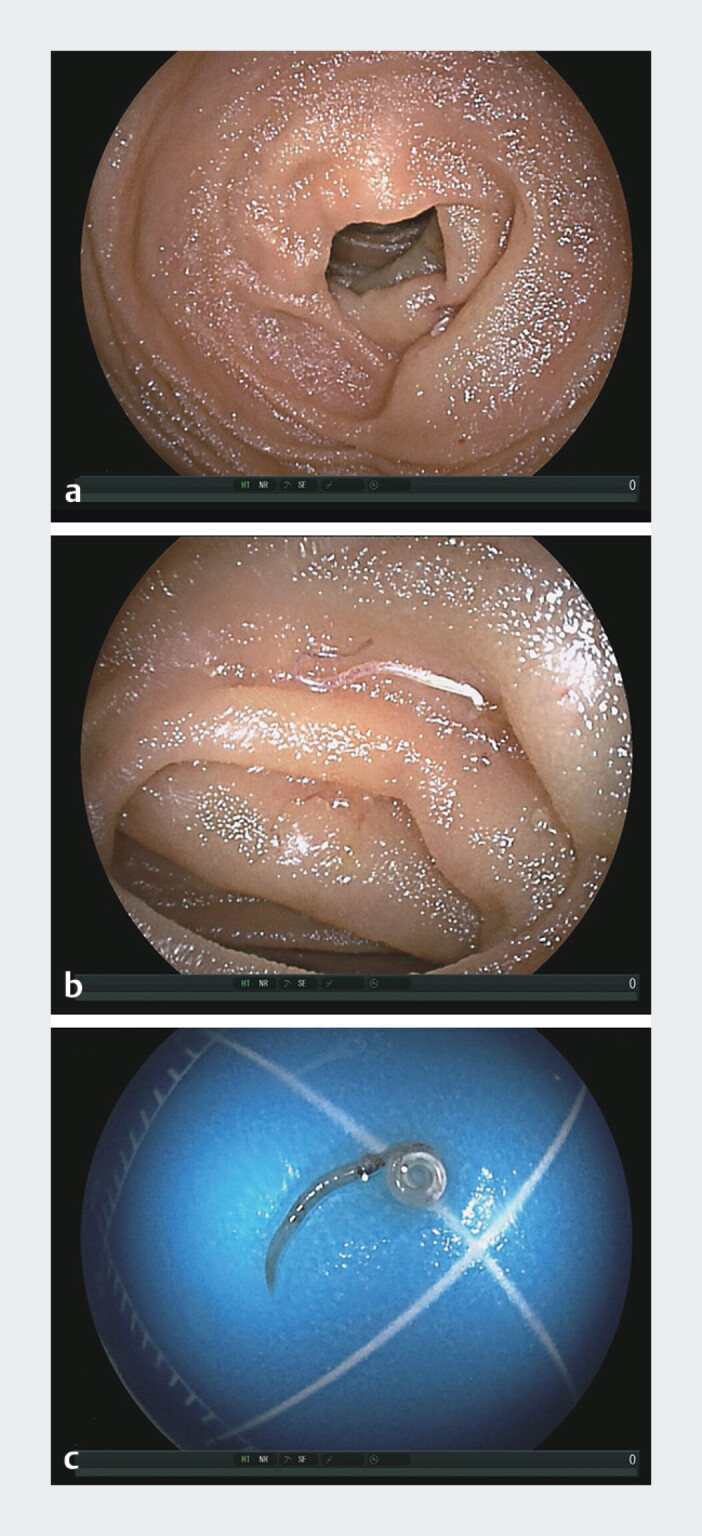
The double-balloon enteroscopy revealed incomplete intestinal obstruction caused by *Enterobius vermicularis.* **a** Intestinal mucosa congestion and edema. **b** The double-balloon enteroscopy revealed an active worm, surrounded by congested and edematous mucosa, with mild bleeding. **c** Worm, adult specimen.

The double-balloon enteroscopy revealed an actively moving worm penetrating the edematous, hyperemic, and slightly bleeding intestinal mucosa, followed by its successful retrieval with biopsy forceps.Video 1

**Fig. 3:**
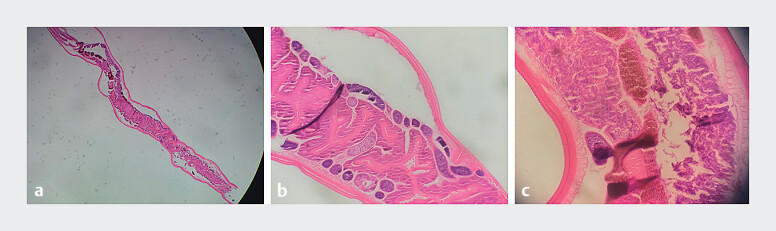
The histopathological analysis confirmed that this specimen was a *Enterobius vermicularis* worm.

Small bowel involvement by *Enterobius vermicularis* is exceptionally rare, particularly when it presents as a mechanical intestinal obstruction. In this case, the diagnosis was established through enteroscopy, which directly visualized a live worm traversing the inflamed mucosa of the ileal segment. Previous reports have documented small bowel obstruction secondary to parasitic infections such as Schistosoma-associated Meckel’s diverticulitis 1 and nematode infestation 2 , both of which were diagnosed intraoperatively through laparoscopy or laparotomy. In contrast, our case highlights the role of deep enteroscopy as a minimally invasive diagnostic and therapeutic modality, representing a rare instance of small bowel obstruction caused by *Enterobius vermicularis* infection that was successfully managed without surgical intervention.

Endoscopy_UCTN_Code_CCL_1AB_2AZ_3AZ

## Notes

Endoscopy E-Videos https://eref.thieme.de/e-videos
*E-Videos* is an open access online section of the journal *Endoscopy* , reporting on interesting cases and new techniques in gastroenterological endoscopy. All papers include a high-quality video and are published with a Creative Commons CC-BY license. Endoscopy E-Videos qualify for HINARI discounts and waivers and eligibility is automatically checked during the submission process. We grant 100% waivers to articles whose corresponding authors are based in Group A countries and 50% waivers to those who are based in Group B countries as classified by Research4Life (see: https://www.research4life.org/access/eligibility/ ). This section has its own submission website at https://mc.manuscriptcentral.com/e-videos .

## References

[1] Almadi F Aljohani E Rare cause of complicated Meckel's with Schistosoma infection: An unusual cause of acute intestinal obstruction in adults Int J Surg Case Rep 2020 66 257 259 10.1016/j.ijscr.2019.11.039 31877547 PMC6931112

[2] Appleby D Kapoor W Karpf M Anisakiasis: nematode infestation producing small-bowel obstruction Arch Surg 1982 117 836 10.1001/archsurg.1982.01380300076016 7200765

